# Efficacy of neoadjuvant chemotherapy combined with surgery in patients with nonsmall cell lung cancer: A meta‐analysis

**DOI:** 10.1111/crj.13756

**Published:** 2024-05-09

**Authors:** Hai‐jun Dong, Cheng‐yan Yang

**Affiliations:** ^1^ Department of Thoracic Surgery, Huzhou Central Hospital Affiliated Central Hospital of Huzhou University Huzhou China; ^2^ Department of Respiratory People's Hospital of Changxing County Huzhou China

**Keywords:** meta‐analysis, neoadjuvant chemotherapy, nonsmall cell lung cancer, surgical treatment

## Abstract

**Introduction:**

This meta‐analysis sought to investigate the effect of neoadjuvant chemotherapy (NACT) combined with surgery in patients with nonsmall cell lung cancer (NSCLC).

**Methods:**

With time span from January 2010 to December 2022, PubMed, Web of Science and Embase, China National Knowledge Infrastructure, and WanFang databases were searched for randomized controlled trials on comparison between NACT combined with surgery and surgery alone in patients with NSCLC. Then a meta‐analysis was performed in accordance with Preferred Reporting Items for Systematic reviews and Meta‐Analyses (PRISMA) guidelines.

**Results:**

A total of 1511 studies were retrieved and 12 were finally included. Meta‐analysis results showed that compared with surgery alone, a combination of NACT and surgery was associated with higher treatment response rate (odds ratio, OR = 2.459, 95% confidence interval, CI [1.785, 3.388], *P* < 0.001), 1‐year survival rate (OR = 2.185, 95% CI [1.608, 2.970], *P* < 0.001), and 3‐year survival rate (OR = 2.195, 95% CI [1.568, 3.073], *P* < 0.001) and lower levels of intraoperative blood loss (standardized mean difference, SMD = −0.932, 95% CI [−1.588, −0.275], *P* = 0.005) and length of hospital stay (SMD = −0.481, 95% CI [−0.933, −0.028], *P* = 0.037).

**Conclusion:**

NACT combined with surgery is superior to surgery alone in the treatment of NSCLC and can promote postoperative recovery. Collectively, such combination is a safe and effective treatment for patients with NSCLC.

## Graphical abstract

This meta‐analysis sought to investigate the effect of neoadjuvant chemotherapy (NACT) combined with surgery in patients with non‐small cell lung cancer (NSCLC). A total of 1511 studies were retrieved by searching the database, and 12 randomized controlled trials (RCTs) were finally included for meta‐analysis. By comparing the clinical outcomes of patients after surgery combined with or without NACT treatment, the results found that NACT combined with surgery was superior to surgery alone in the treatment of NSCLC and promoted postoperative recovery.

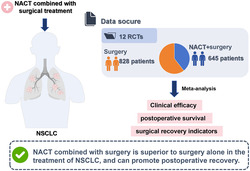

**Abbreviations**
CI: confidence intervals
ESMO: European Society for Medical Oncology
NACT: neoadjuvant chemotherapy
NSCLC: nonsmall cell lung cancer
OR: Odds ratios
PRISMA: Preferred Reporting Items for Systematic reviews and Meta‐Analyses
RCTs: randomized controlled clinical trials
SMDs: standardized mean differences

## 1 INTRODUCTION

Global incidence and mortality of lung cancer have long been higher than that of all cancers.^1^ According to statistics 2018 provided by the International Agency for Research on Cancer, there were 220 000 new lung cancer cases and about 13 500 deaths in the United States, while nonsmall cell lung cancer (NSCLC) represents more than 80% of new diagnoses.^2^, ^3^ Radical surgery is the mainstay of treatment for early stage NSCLC I‐IIIA, but the risk of postoperative recurrence and distant metastasis remains high.^4^ Despite complete resection and adequate adjuvant therapy, approximately 30%–70% of patients will relapse and develop metastases.^5^

The 2017 European Society for Medical Oncology (ESMO) guidelines consider surgical resection (possibly with adjuvant chemotherapy) as the standard of care for patients with early stage NSCLC (UICC stage I and II),^6^ while chemoradiotherapy with or without surgical resection is preferred for patients with locally advanced NSCLC (UICC stage III). Based on current evidence, preoperative chemotherapy should not be routinely used for UICC stage I/II NSCLC, but there is consensus on its effectiveness for UICC stage IIIa (N2) NSCLC, especially for patients with “T downstaging”.^7^ At present, neoadjuvant chemotherapy (NACT) has developed rapidly, which can inactivate tumor tissues and cells metastasizing to regional lymph nodes. As a result, NACT can increase the complete resection rate of surgery, avoiding cancer cell spread, and contributing to scientific judgment of chemosensitivity of patients. More importantly, patients who have no indications for surgery or have potentially resectable tumors get a chance of radical surgery after preoperative NACT.^8^ NACT as a new treatment modality needs to be further confirmed by clinical trials, but reliable clinical data are relatively lacking. So there is an urgent need to assess the available literature to analyze the feasibility, safety, and efficacy of NACT combined with surgical treatment. In this study, we included relevant randomized controlled clinical trials (RCTs) to systematically evaluate the clinical efficacy of NACT combined with surgical treatment in patients with NSCLC.

## 2 MATERIALS AND METHODS

### 2.1 Search strategy

This study was performed in strict accordance with Preferred Reporting Items for Systematic reviews and Meta‐Analyses (PRISMA) guidelines. Two investigators independently searched PubMed, Web of Science, Embase, China National Knowledge Infrastructure, and WanFang databases for collecting relevant RCTs, with the time span from January 2010 to December 2022. Literature search was performed using “neoadjuvant chemotherapy” AND “surgery” AND “nonsmall cell lung cancer OR NSCLC” as search terms.

### 2.2 Inclusion and exclusion criteria

Inclusion criteria are as follows: (1) study subjects: patients diagnosed with NSCLC using histopathological, cytological diagnostic criteria and TNM staging system. (2) study design: studies that had been published in relevant medical journals at home and abroad. (3) intervention measures: The patients were divided into control group and observation group according to different treatment methods. The control group was treated with surgery alone; the observation group was treated with NACT before surgery. (4) Outcome measure (at least including any of the following indicators): treatment response rate, incidence of adverse reactions, 1‐year survival rate after surgery, 3‐year survival rate after surgery, operation time, intraoperative blood loss, and postoperative hospital stay.

Exclusion criteria are s follows: (1) intervention measures or study subjects were not met the requirement of this meta‐analysis, (2) non‐RCTs, (3) duplicate articles, and (4) original study providing no data required for this meta‐analysis.

### 2.3 Literature screening and data extraction

The obtained data were imported into Endnote7.0 software and duplicate literature was eliminated. Two investigators independently screened literature according to the predesigned inclusion and exclusion criteria, followed by data extraction and evaluation of the quality. In case of disagreements, a consensus was reached through discussing with a third investigator. Relevant data extracted included are as follows: article title, first author, study design, interventions, patient baseline data, and outcome measures. In addition, the Cochrane Collaboration's RCTs tool was used to assess the risk of bias included in the meta‐analysis.

### 2.4 Statistical analysis

Meta‐analysis was performed using Stata 16.0 statistical software. Odds ratios (OR) and 95% confidence intervals (CI) were used to describe dichotomous variables and standardized mean differences (SMDs) and 95% CI to present continuous variables. Heterogeneity among studies was assessed by *Q* test and *I* ^2^ test. If *P* > 0.05 and *I* ^2^ < 50%, there was no significant heterogeneity among studies, and fixed‐effects model was employed for statistical analysis; otherwise, random‐effects model was used. Further, sensitivity analysis was carried out to identify the source of heterogeneity.

## 3 RESULTS

### 3.1 Basic information of included studies

**FIGURE 1:**
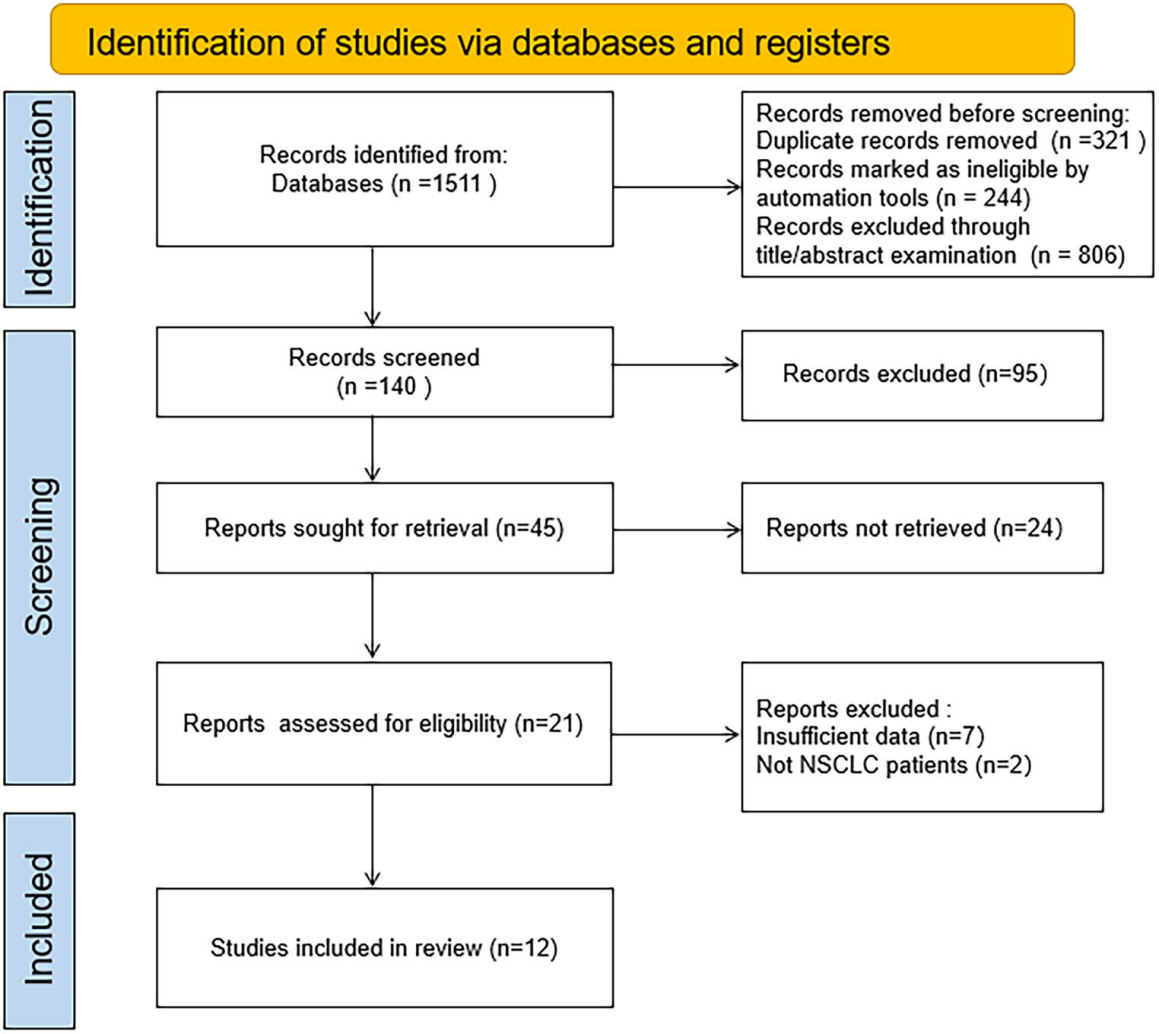
Flow chart of literature screening.

**FIGURE 2:**
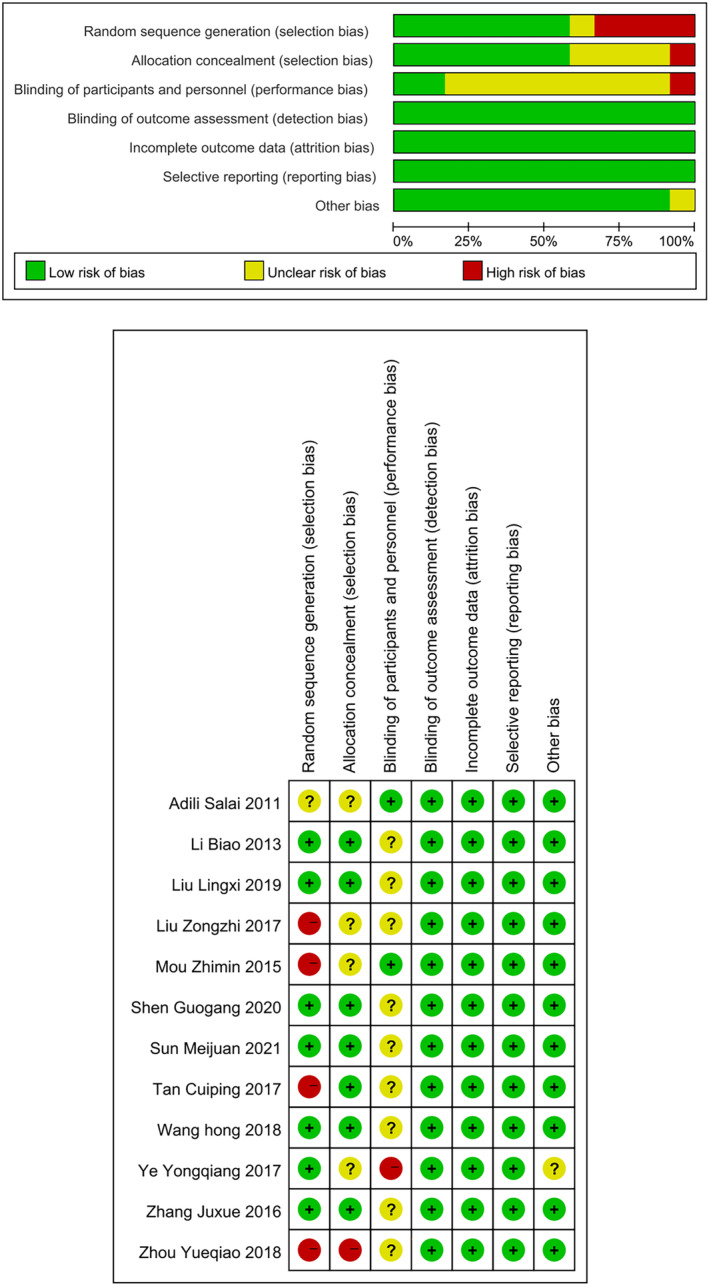
The risk of bias graph and summary.

A total of 1511 literature were retrieved in this study, and then 321 duplicate articles and 1050 unqualified articles were preliminarily excluded. After full‐text review, 128 literature was then removed, and finally 12 RCTs^9^, ^10^, ^11^, ^12^, ^13^, ^14^, ^15^, ^16^, ^17^, ^18^, ^19^, ^20^ were included for meta‐analysis. The flow chart of literature screening is shown in Figure 1. A total of 1473 patients were included in this study, with 645 patients in the observation group (NACT combined with surgery) and 828 patients in the control group (surgery alone). The characteristics of each included study are shown in Table 1. As shown in Figure 2, no risk of bias was found for the included RCTs in our meta‐analysis.

**TABLE 1:** Basic characteristics of included literature.

Study	Year	Sample time	Clinical stages	Group	Cases	Age (years)	M/cases	Study design	Outcome measures
Ye Yongqiang	2017	2012/06 to 2016/06	IIIa ‐N2	Obse	17	56.67 ± 9.53	13	RCT	①②⑤⑥⑦
				Cont	20	54.33 ± 11.26	16		
Mou Zhimin	2015	2008/01 to 2011/06	IIIa	Obse	30	58.4 ± 11.3	18	RCT	①②③④⑤⑥
				Cont	25	57.2 ± 10.9	14		
Tan Cuiping	2017	2011/01 to 2013/10	IIIa	Obse	97	36–73	75	RCT	②⑤⑥⑦
				Cont	273	20–78	207		
Sun Meijuan	2021	2017/02 to 2018/12	I, II, IIIa	Obse	43	59.86 ± 6.67	28	RCT	①②③⑤⑥⑦
				Cont	43	60.28 ± 6.25	31		
Liu Lingxi	2019	2015/01 to 2016/12	/	Obse	30	65.34 ± 5.23	20	RCT	①②③⑤⑥⑦
				Cont	30	65.39 ± 5.12	21		
Shen Guogang	2020	2014/04 to 2018/12	/	Obse	25	65.28 ± 7.7	15	RCT	①③④
				Cont	25	65.34 ± 8.45	16		
Zhang Juxue	2016	2009/12 to 2012/12	IIIa, IIIb	Obse	36	57.1 ± 4.8	23	RCT	①③④⑤⑥
				Cont	30	59.4 ± 5.1	20		
Adili Salai	2011	2001/01 to 2004/12	IIIa, IIIb	Obse	59	60.19 ± 9.15	30	RCT	①②③④⑤
				Cont	60	58.31 ± 9.74	35		
Zhou Yueqiao	2018	2015/01 to 2016/12	IIIa	Obse	150	47.98 ± 3.71	97	RCT	①②③⑤⑥⑦
				Cont	150	48.75 ± 3.56	92		
Liu Zongzhi	2017	2010/05 to 2014/09	IIIa, IIIb	Obse	43	56.28 ± 8.12	23	RCT	②③④
				Cont	57	55.97 ± 9.05	25		
Li Biao	2013	2006/12 to 2009/12	IIIa, IIIb	Obse	55	56.8 ± 4.5	35	RCT	①②③④⑤⑥
				Cont	55	57.2 ± 5.2	36		
Wang hong	2018	2010/07 to 2012/06	IIIa, IIIb	Obse	60	63.76 ± 6.43	36	RCT	②③④⑤⑥
				Cont	60	63.82 ± 6.49	39		

*Note*: ①: effective rate after treatment; ②: incidence of adverse reactions; ③: 1‐year survival rate after surgery; ④: 3‐year survival rate after surgery; ⑤: operation time of operation; ⑥: intraoperatve blood loss; ⑦: postoperative hospital stay.

Abbreviations: Con, control group; M, male; Obse, observation group; RCT, randomized controlled trial.

### 3.2 Meta‐analysis of clinical efficacy in patients after treatment

**FIGURE 3:**
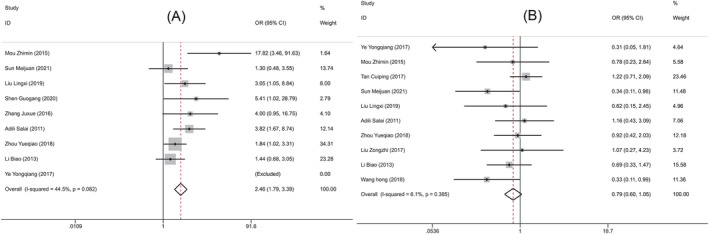
Forest plots comparing the clinical efficacy of two groups of patient with nonsmall lung cancer. (A) Forest plot of treatment response rate. (B) Forest plot of incidence of adverse reactions.

**FIGURE 4:**
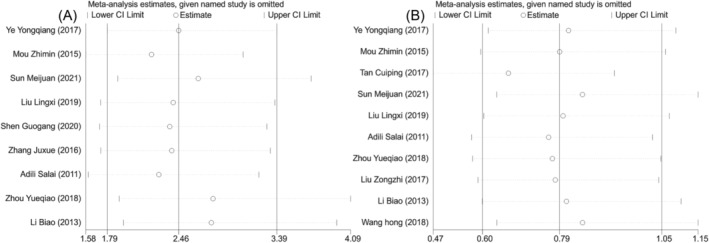
Sensitivity analysis of clinical efficacy of two groups of patients with nonsmall cell lung cancer. (A) Sensitivity analysis of treatment response rate. (B) Sensitivity analysis of adverse reactions.

Nine RCTs^9^, ^10^, ^12^, ^13^, ^14^, ^15^, ^16^, ^17^, ^18^ including 883 patients and 10^9^, ^10^, ^11^, ^12^, ^13^, ^16^, ^17^, ^18^, ^19^, ^20^ studies including 1357 cases compared the response rate and the incidence of adverse reactions between the two groups of patients, respectively. Fixed‐effects model was used to pool effect sizes due to presence of significant heterogeneity (treatment response rate: *P* = 0.082, *I* ^2^ = 44.5%; incidence of adverse reactions: *P* = 0.385, *I* ^2^ = 6.1%). Meta‐analysis results showed that compared with surgery alone, a combination of NACT and surgery was associated with higher treatment response rate (OR = 2.459, 95% CI [1.785, 3.388], *P* < 0.001, Figure 3A). However, there was no significant difference in the incidence of adverse reactions between the two groups (OR = 0.792, 95% CI [0.595, 1.054], *P* = 0.11, Figure 3B). Further sensitivity analysis was performed. After one‐by‐one removal, it was found that the heterogeneity of treatment response rate might be attributed to the studies by Mou et al.,^10^ Zhou et al.,^17^ and Li et al.,^19^ and the study by Tang^11^ might be responsible for the heterogeneity of incidence rate of adverse reactions. But there was no effect on the results (Figure 4A,B). Collectively, the meta‐analysis results of this study were relatively stable and reliable.

### 3.3 Meta‐analysis of postoperative survival

**FIGURE 5:**
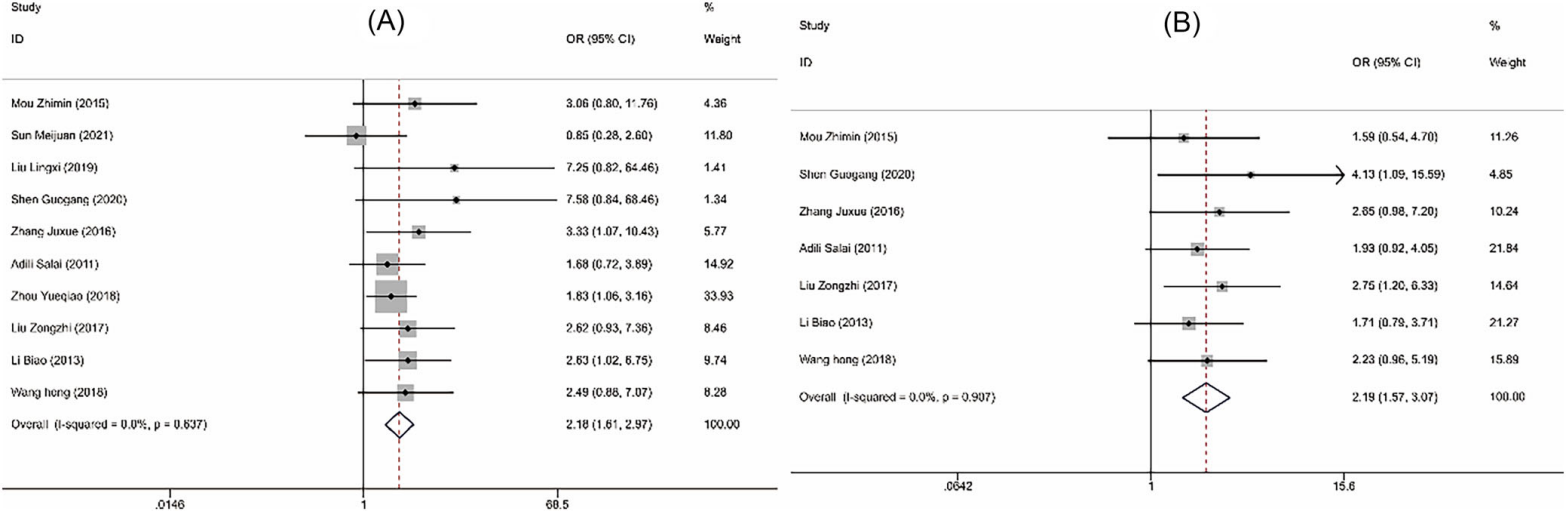
Forest plots comparing postoperative survival of two groups of patients. (A) Forest plot of 1‐year survival rate. (B) Forest plot of 3‐year survival rate.

**FIGURE 6:**
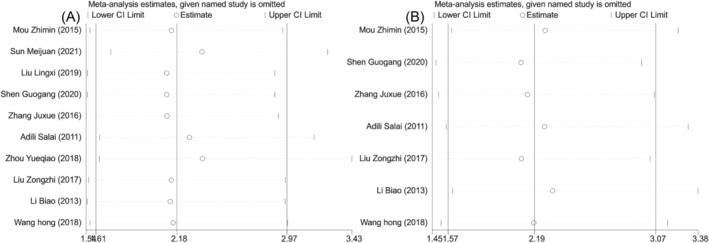
Sensitivity analysis of postoperative survival. (A) Sensitivity analysis of 1‐year survival rate. (B) Sensitivity analysis of 3‐year survival rate.

Ten RCTs^10^, ^12^, ^13^, ^14^, ^15^, ^16^, ^17^, ^18^, ^19^, ^20^ including 1066 patients and 7^10^, ^14^, ^15^, ^16^, ^18^, ^19^, ^20^ studies including 620 patients compared the 1‐year survival rate and 3‐year survival rate after surgery between the two groups of patients, respectively. Fixed‐effects model was employed for pooling effect sizes because of no heterogeneity among the included studies (1‐year survival rate after surgery: *P* = 0.637, *I* ^2^ = 0.0%; 3‐year survival rate after surgery: *P* = 0.907, *I* ^2^ = 0.0%). Meta‐analysis results exhibited that the 1‐year survival rate (OR = 2.185, 95% CI [1.608, 2.970], *P* < 0.001; Figure 5A) and 3‐year survival rate (OR = 2.195, 95% CI [1.568, 3.073], *P* < 0.001; Figure 5B) in the observation group were significantly higher than those in the control group. In sensitivity analysis, the pooled results newly obtained after one‐by‐one removal still showed that the 1‐year survival rate and 3‐year survival rate in the observation group were better than those in the control group. That meant that the results of this study were relatively stable and reliable (Figure 6A,B).

### 3.4 Meta‐analysis of surgical recovery indicators

**FIGURE 7:**
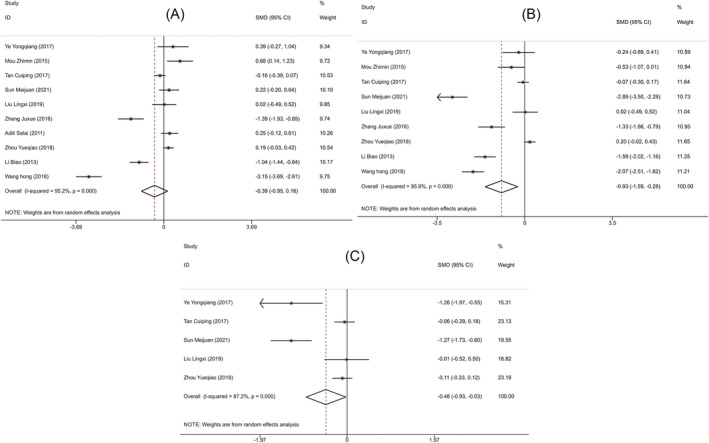
Forest plots comparing surgical recovery indicators in two groups of patients. (A) Forest plot of operation time. (B) Forest plot of intraoperative blood loss. (C) Forest plot of hospital stay.

**FIGURE 8:**
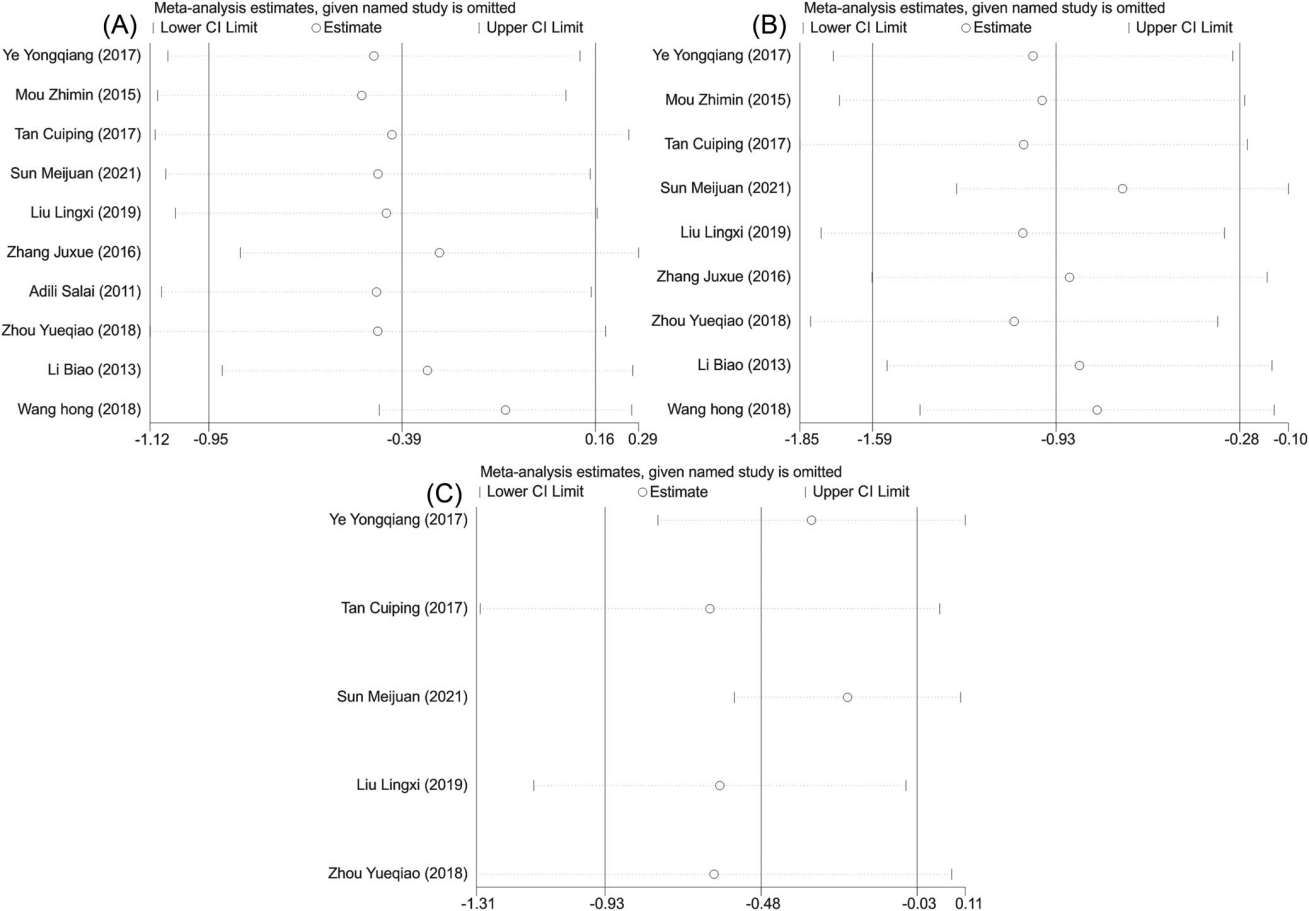
Sensitivity analysis of surgical recovery indicators in two groups of patients. (A) Sensitivity analysis of operation time. (B) Sensitivity analysis of intraoperative blood loss. (C) Sensitivity analysis of hospital stay.

Ten RCTs^9^, ^10^, ^11^, ^12^, ^13^, ^15^, ^16^, ^17^, ^19^, ^20^ including 1323 patients, 9^9^, ^10^, ^11^, ^12^, ^13^, ^15^, ^17^, ^19^, ^20^ studies including 1204 patients, and 5^9^, ^11^, ^12^, ^13^, ^17^ studies including 853 patients compared the operation time, intraoperative blood loss, and hospital stay between the two groups, respectively. And random‐effects model was used to pool effect sizes owing to significant heterogeneity (operation time: *P* < 0.001, *I* ^2^ = 95.2%; intraoperative blood loss: *P* < 0.001, *I* ^2^ = 95.9%; hospital stay: *P* < 0.01, *I* ^2^ = 87.2%) (Figure 7A–C). According to the meta‐analysis results, the observation group had lower levels of intraoperative blood loss (SMD = −0.932, 95% CI [−1.588, −0.275], *P* = 0.005, Figure 7B) and hospital stay (SMD = −0.481, 95% CI [−0.933, −0.028], *P* = 0.037; Figure 7C) compared with the control group. But there was no significant difference in operation time (SMD = −0.392, 95% CI [−0.947, 0.162], *P* = 0.166; Figure 7A) between the two groups. Further sensitivity analysis using one‐by‐one removal method, the studies by Wang et al.,^20^ and Sun and Wang^12^ might be responsible for the heterogeneity across included studies in treatment operation time, intraoperative blood loss, and hospital stay. But the newly obtained results remained unchanged, suggesting that the results of this study were relatively stable and reliable (Figure 8A–C).

## 4 DISCUSSION

NSCLC, as a cancer with high morbidity and mortality, seriously endangers people's health and quality of life. Studies have demonstrated that NACT combined with surgical treatment plays a crucial role in improving the prognosis of patients with lung cancer.^21^ And NACT and neoadjuvant radiotherapy as systemic have been recommended by the National Comprehensive Cancer Network (NCCN) guidelines.^22^ According to previous studies and NCCN guidelines, postoperative adjuvant cisplatin‐based chemotherapy has become the standard of care for patients with completely resected high‐risk stage IB and II‐IIIA NSCLC.^23^ A meta‐analysis of 13 RCTs also proved that NACT significantly improved overall survival in NSCLC patients with III stage compared with surgery alone.^24^ A combination of NACT and surgery may be a better option for patients with early resectable NSCLC.^25^, ^26^, ^27^

In this study, the meta‐analysis we collected from the relevant published literature at home and abroad and results of 12 studies were combined and analyzed to increase the sample size and improve the power of the test. As a result, the obtained pooled results were more reliable. Specifically, meta‐analysis showed that compared with surgery alone, NACT combined with surgery in the treatment of patients with NSCLC improved the treatment response rate, which is consistent with relevant studies.^28^ Also, such combination effectively shortened the hospital stay and intraoperative blood loss. Previous studies have demonstrated that operation time >3 h, hospital stay >30 days, and invasive procedures are risk factors affecting postoperative nosocomial infection.^29^ In other words, increased operative time, length of hospital stay, and intraoperative blood loss are not only a reflection of the patient's severity of illness and also increase the risk of infection in patients with NSCLC, resulting in adverse clinical outcomes.

One of the main criteria for the efficacy of lung cancer treatment is whether it can prolong the survival of patients. According to the 2020 American Society of Clinical Oncology (ASCO) statistics, the 5‐year survival rate for NSCLC is 24% and <5% for patients with locally advanced or metastatic NSCLC.^30^ Clinical data in China showed that the 1‐, 2‐, and 3‐year survival rates of patients with NSCLC were 83.4%, 47.4%, and 33.4%, respectively.^31^ The results of this meta‐analysis revealed that NACT could significantly improve the 1‐ and 3‐year survival rate of patients with NSCLC and prolong survival. This is because NACT combined with surgical therapy not only inhibits the recurrence of incompletely resected primary tumors but also avoids the occurrence of potential occult micrometastases outside the lung.^32^, ^33^ So NACT combined with surgical treatment can be selected in order to obtain a better therapeutic effect under full consideration of the patient's cancer stage and physical tolerance.

In summary, the meta‐analysis suggests that for patients with NSCLC, NACT combined with surgery has higher efficacy and safety compared with surgery alone. However, this study still has some limitations. First, all included studies in this study are Chines, and the stability of meta‐analysis results was limited by the number and quality of included studies. It is possible that this is the reason why we limited the literature search to the years 2010 to 2022. Second, the small amount of included literature failed to perform a subgroup analysis of adjuvant chemotherapy regimens, which can lead to some bias in the results. Further, large‐sample, multicenter RCTs are still needed to enhance the accuracy and reliability of the study results, in order to provide a more effective method for the next step of clinical treatment of NSCLC.

## AUTHOR CONTRIBUTIONS

Hai‐jun Dong designed this work. Cheng‐yan Yang searched the articles. Hai‐jun Dong and Cheng‐yan Yang performed the data extraction, statistical analyses, and wrote this article. All authors read and approved the final manuscript.

## CONFLICT OF INTEREST STATEMENT

The authors declare that they have no competing interests.

## ETHICS STATEMENT

This is a meta‐analysis and ethics approval and consent to participate are not required.

## Data Availability

The data that support the findings of this study are available from the corresponding author upon reasonable request.
